# A report on the LASFs 3rd Annual Conference on the Validation of Laboratory Systems

**DOI:** 10.1155/S1463924696000089

**Published:** 1996

**Authors:** Joseph G. Liscouski

**Affiliations:** Laboratory Automation Standards Foundation (LASF) PO Box 38 Groton MA 01450 USA


Journal of Automatic Chemistry, Vol. 18, No. 3 (May–June 1996), pp. 115–119

### A report on the LASFs 3rd Annual Conference on the Validation of Laboratory Systems

**Joseph G. Liscouski**
*Laboratory Automation Standards Foundation (LASF), PO Box 38, Groton, MA 01450, USA*

*The purpose of the Foundation's conferences on systems validation is to examine topics in depth. The 1994 meeting covered the Validation of Laboratory Information Management Systems [1]. The 1995 meeting was concerned with:*

*(1) Conducting vendor audits—important because of the FDA's consideration of adding a vendor audit requirement to GMPs/GLPs.*
*(2) Validation of data acquisition systems.*
*(3) The impact of data interchange standards on systems validation.*

*Each of the sessions consisted of presentations followed by an open forum of questions and discussion.*

### Conducting vendor audits

The purpose of a *vendor audit* is to evaluate a potential supplier's ability to produce and support a product. Producing a product involves the ability to understand user requirements, to turn those requirements into a functional specification and then into a usable product. Support covers everything from long-term financial stability, answering questions about products, providing a forum for customer feedback, to enhancement of a product to meet changing requirements.

Audits are an important part of the product evaluation/acquisition process. As a result of purchasing a product you are entrusting some aspect of your company's business potential to that product, be it an instrument, computer, or software system. Before doing this, you need to be sure that the vendor is capable of delivering a reliable product and maintaining that product over its useful life. Given the rash of corporate mergers, down-sizing or right-sizing, changing technology, and, changes in management perspectives in the industry, a company's ability to support a product can vary considerably over time.

Vendor audits are not a simple process. People need to be able to evaluate the technical competence and financial health of a vendor, as well as its commitment to a product line. As a result, the audit team should have competence in systems engineering, quality management, the underlying science in a product, and financial evaluations.

The meeting was structured to provide an understanding of key points in a vendor audit and then to discuss them in detail. Presentations included:

(*a*) *The development of software applications*—Michael Dudek (WMX Environmental Monitoring Laboratories) discussed current software engineering practices and how they should be used in product development.

(*b*) *The current status of software supplier certification*—Ken Chapman (Drumbeat Dimensions) covered the current status of ISO-9000-3 and TickIT evaluations. The UK's GMP guidelines and the PDA's Technical Report No. 18 were also covered.

(*c*) *Vendor audits: GMPs versus ISO-9000*—by John Read (Laboratory Management Systems) examined the differences and common elements of approaches by regulated and non-regulated companies.

(*d*) *Evaluation of software quality*—Bruce Katz (Software Quality Assurance) reviewed the strategies used for software testing, its role in software development, and the development of metrics for software quality.

(*e*) *The role of ISO 9000 and ISO 14001 in vendor audits*—Andrew Rowe (National Quality Assurance) and Samantha Munn (Inchcape Testing Services/Intertek Services) discussed the details of these standards and their role in evaluating vendor quality and management practices.

(*f*) *Vendor audits from a vendor's perspective*—was covered in presentations by Gary Burce (Varian), William Maxwell (Waters Corporation), Fernando Casanova (Beckman) and Jo Webber (LabWare).

(*g*) *Vendor audits from a user's view*—was discussed by George Grigonis (Merck & Co.).

(*h*) *Software vendor inspection: an FDA investigator's perspective* was given by Dave Bergeson of the US Food and Drug Administration.

There were two key questions that the Foundation wanted to address: How do you conduct an audit? And is there a role that third party organizations can play in making the vendor audit more cost-effective and efficient? During the 1994 meeting Paul Motise stated that ISO-9000 certification would not be acceptable to the FDA, since the FDA has no authority over ISO auditors, and has no way of ensuring that auditors are qualified in the requirements of FDA regulated industries.

### *How to conduct an audit*

One of the major subjects under discussion was what should be covered during a vendor audit. For much of

0142-0453/96 $12.00 © 1996 Taylor & Francis Ltd.

the discussion, it was assumed that a product existed and had been evaluated as suitable for a given purpose. Dr Grigonis proposed an initial outline of evaluation topics, which during the open forum was modified to this:

(0) *Preparation*: Be prepared for the visit—the trip is an expense to both the user and vendor. Prior to the visit make sure that the vendor knows what your purpose is and what you want to see. Give the vendor a detailed list of materials that you want to evaluate—some can be provided to you before the actual visit in printed form shortening the time on-site (a non-disclosure agreement may be required). The duration of the visit should be reasonable: a few days at most.

(1) *Software development methods*: How does the vendor address the following:
- Software development process—what programming standards are used, are they well defined and followed?
- Reusable software products—if software is reused over time and different products, is that software kept up to current development practices? Is it well maintained? (Reusing software can add efficiency to the development process, but 'old' software may not be well documented or maintained. Some old code may also be part of product prototypes and not up to commercial standards.)
- Purchased products and services—what criteria are used to evaluate vendors and products?
- Source code—what are the company's practices for making source code available?

(2) *Software maintenance practices*:
- How does the vendor respond to bug reports?
- Are fixes distributed on a monthly, quarterly, or other basis, or do they arrive with incremental releases?
- Do you have to be part of a maintenance programme to get bug fixes?
- How long is the warrantee period? When does it begin?

(3) *Software testing practices*:
- What are the vendors criteria for releasing a product?
- What are the policies for entry and exit to/from beta test?
- What are the criteria for being part of a beta test program?

(4) *Software manufacturing practices*:
- How are distributed versions verified?
- How does the vendor ensure that distributed copies are virus free?
- How are released versions keyed to releases of underlying operating systems and/or language releases?

*Note: The same points noted above for software can be translated to hardware and documentation. The numbered headings are listed but the details are not, they are essentially an intelligent 'ditto' from their software counterparts.*

(5) *Hardware engineering practices.*

(6) *Hardware maintenance practices.*

(7) *Hardware testing practices.*

(8) *Hardware manufacturing practices.*

(9) *User documentation development.*

(10) *User documentation maintenance.*

(11) *Related practices*:
- Training—what courses are available? Are they only given at the vendor site or are can they be given at the customer site?
- Developer training—are the people doing product development well schooled in their discipline? What evidence is there?
- Configuration management—what constitutes the current vendor recommended mix of hardware and software (as appropriate)? For PC systems, is there a required version of the BIOS?
- Version management.
- Documentation management.
- Supplier or subcontractor management—is ISO 9000 certification a requirement for suppliers?
- Quality assurance.

(12) *Quality management systems*:
- What is the vendor's quality management programme? How is it maintained, evaluated, and enforced? Is the vendor ISO 9000 certified? If a software product is involved, is it TickIT certified? If not, why? Is continuous improvement part of the process?

(13) *Contracted services*:
- How are contractors evaluated and chosen?
- How are their products tested before being accepted?
- Are critical components of products outsourced?
- How are the products maintained?
- Who owns the contracted products and maintains source control?
- How is quality maintained?

(14) *Evaluation questionnaire*:
- Prior to the visit you should determine the key points you want addressed by any vendor, these form the basic questionnaire.
- Based on a review of documentation supplied by the vendor, what additional information do you require?

(15) *Business issues:*
- Is the product profitable (products that loose money don't survive long)?
- Is the company profitable?
- Is it growing?
- Is the company's business dependent upon the product? If it isn't tied to their main-line business, will it get the attention you want it to have?
- What is the size of the installed base, and is it growing?
- Is there an independent user-group? Does the company participate?

- Is there a BBS for questions and support?
- Can you get a walk-through of the company? Seeing the workplace will give you an idea of employee attitudes, work load, and corporate health (graffiti on the walls is not a good sign, neither are cartoons of management, or screen savers that say 'just go away'). If you get the impression that you wouldn't want to work at a company, do you want to buy from them?

Ideally, every product/service vendor should be audited, but that isn't realistic. Laboratory instrument and software vendors are open to the process, but second-tier vendors (operating systems, computer hardware) would not be. In practical terms, their business wouldn't suffer enough by not giving you a chance to audit their company. For these suppliers you do have some recourse.

The vendors you are buying software and hardware from are choosing the platforms and systems to be used, either by directly providing them or by making recommendations. How are those recommendations arrived at? How have those choices been tested and evaluated (see items 1, 5, and 13 above)? In addition, there is a large population of users of popular hardware and software—what are their experiences? Check BBSs and user groups, including local chapters of computing societies.

The make-up of the audit team is critical. Make sure that people have the backgrounds and experience needed to evaluate the topics under consideration. Use outside consultants to fill in gaps. Consider audit guidelines produced by other organizations, the ASQC was noted as one useful source.

The vendors at the meeting noted that they are rarely audited more than once by their customers. Some users noted that their policies require periodic audit updates. You are responsible for your own audit policies. They should be guided by more than the calendar. Have the company's business practices changed? How has their financial condition changed? Are their shifts in senior management? Are mergers, acquisitions, layoffs, or sales of product lines a factor? Any significant business or technology event should trigger a review of a vendor. That review process may either be substantial or a minor update depending on the nature of the event.

*Is there a role for third-party audits?*

The consensus is yes. If you examine the list above, much of it is common to the companies in a given industry. Third parties could be a useful *supplement* to your own audits, reducing the time on-site and the cost of the audit process. Some of the attending companies noted that they have used contractors to perform some of the audit activities.

There are issues that need to be worked, but they are workable. ISO-9000 as it stands will not suffice. The issues here are: acceptance of the auditors' work by the FDA; accountability of auditors to regulating agencies; whether the auditors are qualified to evaluate vendors of laboratory instruments and systems; and ISO-9000 is an evaluation of management practices, not products. Similar issues have been addressed in the aircraft and automotive industries. QS-9000, for example, is the automotive add-on/upgrade of ISO-9000, designed to address industry specific requirements. Who is the appropriate group to create the laboratory equivalent of QS-9000? Creating a separate version for pharmaceutical, biotechnology, environmental, clinical and others would be a mistake. There aren't enough differences to justify overlapping requirements. The variations can be handled by the customer portion of the audit—remember the proposal is that third-party audits are part of your audit process, not a complete replacement for it.

Once requirements are determined, registrars would have to be qualified before they could conduct an audit, and their work would be checked periodically. This is where the accountability to regulating agencies could take place.

This is an area where the scientific community needs to take an active role and determine its own course of action, rather than wait for that course to be dictated. Products are becoming more complex and business practices are changing. More efficient methods of evaluating vendors is needed to reduce costs while maintaining or improving reliability.

### Validation of data acquisition systems

The validation of data systems is becoming increasingly complex. The software for off-the-shelf packages does more and is beginning to overlap many traditional functions that had been relegated to Laboratory Information Management Systems. Operating systems have changed, allowing users to execute more than one function on a computer at the same time with the serious potential of compromising data acquisition. Operating system modifiers (speed doublers, RAM doublers, disk compression) are available from third parties, all of which have the potential for disrupting the behaviour of lab systems.

In addition, the software tools for developing data systems has moved to the use of graphical user interfaces (GUI). Developers can create an entire system without writing a line of traditional programming code; a model that is moving into data-base applications.

Three presentations were made on this topic:

(1) *Utilizing standards in PC-based data acquisition test systems*, by Chad Stalker (Data Translation).

(2) *Validating programming to meet quality standards*, by Ed Kruft (National Instruments).

(3) *A chromatographic simulator for computer validation of chromatography systems*, by Ray Miller (PharmCom).

During the discussion phase of the session—which covered a wide range of topics—a number of points were raised:

(*a*) PCs using the ISA bus were less reliable than PCI or PCMCIA based systems—it is possible to loose data on ISA bus systems.

(*b*) The increasing use of Rapid Application Development systems does not replace the need for proper documentation of user requirements, and software/hardware engineering specifications.

(*c*) Problems have been noted in repairing and upgrading PC systems—repaired/upgraded systems may not function properly in data acquisition applications. Hardware changes should be considered as a reason for system re-validation, not just hardware qualification. Although the hardware may function properly according to vendor diagnostic routines, the interaction of data acquisition software and hardware may be more stressful than the diagnostics.

(*d*) People need to be well trained in the use of systems, not just the sequence of buttons to push, but to recognize potential failures, and deviations from expected behaviour. We need to resist the temptation to accept the output of data systems at face value and to be skeptical of results.

(*e*) Users requested that the vendors identify all critical system files used (DLLs, system 'in' files, etc.), so that care can be taken to prevent other applications from compromising these resources.

One discussion covered concerns in validating modules used in GUI systems, like National Instrument's LABView. Functional testing can be done, but structural validation cannot since the source code is not provided by the vendor. Ed Kruft noted that this situation is no different than developers using subroutine libraries for data acquisition, graphics, or database systems. The vendor audit should determine whether or not a vendor is using good software engineering practices (GSEP). The experience of users (gained through participation in user groups for example) is a means of gaining confidence in a vendor's products—similar to the acceptance of operating systems, languages, and large-scale programming systems. Reliable vendors should be providing bug reports and fixes for software—software maintenance programs are a good way of keeping abreast of developments and fixes for problems. It was also pointed out that the validation of a system is version specific. If a programming module is updated to a new version, the system should be re-validated before being put to use.

Ray Miller's presentation raised considerable interest. Among the items covered were the behaviour of systems after power-failure, noise rejection, and the effect of peak-height ratio on integration accuracy. The power-fail issue is an interesting problem. Having systems operate through a power-failure is an out-of-the-ordinary situation, and the data captured should be considered suspect and samples re-run. Power systems are under increasing stress, and forecasts for the next few years indicate that demand will out-strip supply. While 100% power loss may not be commonplace, low-voltages (brown-outs) can trip power-fail conditions *without your being aware of it*—and they are becoming common. Power surges from lightning are an issue as well. This testing is significant.

Some of the systems tested did not fair well through simulated power-fail conditions. Sample data files did not match the samples. This does not necessarily point up a flaw in the design of the software system, but rather a consideration that must be taken into account in the design of automated laboratories—provide backup power supplies for computer systems to help them survive brown-outs and to do a clean shut-down during black-outs. Then test them.

Audio-tapes of these sessions are available, contact the LASF for more information.

### Data interchange standards and regulatory requirements

This session dealt with an important issue in the use of automation within regulated industries: the introduction of new technologies. Software and hardware technology has changed considerably since the 1970s when the FDA GLPs and GMPs were drafted. The development of PCs, commercial software, and data interchange standards are just a few of the changes that have taken place in the last 17 years.

In 1992 the Analytical Instrument Association (AIA) released the first draft of the 'andi' Chromatography Standard [2]. This software represented the initial attempt to provide data in a vendor interchangeable format. The ability to export data in a format that can be easily read by other software opens up the possibilities of long-term archiving, vendor independence, protection for data over long time-frames, and the ability to use software packages from multiple sources on the same data set. All of the features that make office automation and desktop publishing systems viable are denied to laboratory automation systems. These points were well summarized by Glen Wollenburg (Merck & Co.) during his introduction to the session, when he covered the current state of the standards, their potential, and problems:

(1) Data has to be accessible over long periods of time; at least for a decade and perhaps for as long as 18 years. This is longer than products survive, and longer than many companies endure. Suppose the vendor for your data system goes out of business or is acquired by another company and that data system product is dropped. In 10 years you may see five generations of computer technology. Unless platform and vendor independant standards are implemented you may not be able to read your data.

(2) When you purchase a system, the customer/vendor relationship is similar to a marriage contract—the vendor holds your data in a format that they control with access only through their software.

(3) If a company wants to change data system vendors, they still have to maintain a legacy system to permit access to older data.

The work started by the AIA has a number of potential benefits. But there are problems. For instance, the current definition of 'original raw data' does not permit the transformation of data in one file format to another, and having that second file still be considered primary raw data. This is a major limitation to the development and implementation of advanced systems. It prevents vendors from taking full advantage of new database technologies, since they have to support the old data formats—the regulations prevent them from transforming data formats as well as the user.

The development of the standards is not proceeding quickly enough. Old versions of standards need to be updated to reflect current customer needs, including GLP/GMP compliance information. The standards need to be more broadly implemented. The user community needs to become more actively involved in standards specification.

The point of the session was not to solve the problems, but to begin identifying them. Part of the solution is going to require a change in the regulations, bringing them up to at least current technology and ideally providing guidance for the adoption of new technologies. Part of it also relies on the vendor and user communities. If vendors adopted the standard file formats as their primary storage format, the definition of raw data would cease to be an issue. Users need to be more active and vocal in the standards development process and then demand their adoption.

Of all the points, the latter is the most significant. Without user pressure, nothing will happen. Vendors respond to customer demand. Lacking that demand, no changes will take place. An attempt was made in the Spring of 1995 to organize a conference on the standards issue. It didn't happen due to lack of registrations. The poor response was at least in part due to a perception that standards are a long-term issue. That 'long-term' issue in now eight months (at this writing) closer.

### References

1. Richter, M., *American Clinical Laboratory* (April 1995).
2. Liscouski, J.G., *Laboratory and Scientific Computing: A Strategic Approach* (John Wiley & Sons, New York, 1995).

*For more information about this meeting, obtaining copies of the audio-tapes, or about future sessions, contact the LASF at PO Box 38, Groton, MA 01450, telephone at 508 448 6130 or EMail (joe166@aol.com).*

